# Flexible Piezoresistive Sensors Embedded in 3D Printed Tires

**DOI:** 10.3390/s17030656

**Published:** 2017-03-22

**Authors:** Md Omar Faruk Emon, Jae-Won Choi

**Affiliations:** Department of Mechanical Engineering, The University of Akron, Akron, OH 44313, USA; me49@zips.uakron.edu

**Keywords:** tire sensor, tactile sensing, piezoresistive sensor, 3D printing, road condition, tire condition

## Abstract

In this article, we report the development of a flexible, 3D printable piezoresistive pressure sensor capable of measuring force and detecting the location of the force. The multilayer sensor comprises of an ionic liquid-based piezoresistive intermediate layer in between carbon nanotube (CNT)-based stretchable electrodes. A sensor containing an array of different sensing units was embedded on the inner liner surface of a 3D printed tire to provide with force information at different points of contact between the tire and road. Four scaled tires, as well as wheels, were 3D printed using a flexible and a rigid material, respectively, which were later assembled with a 3D-printed chassis. Only one tire was equipped with a sensor and the chassis was driven through a motorized linear stage at different speeds and load conditions to evaluate the sensor performance. The sensor was fabricated via molding and screen printing processes using a commercially available 3D-printable photopolymer as 3D printing is our target manufacturing technique to fabricate the entire tire assembly with the sensor. Results show that the proposed sensors, inserted in the 3D printed tire assembly, could detect forces, as well as their locations, properly.

## 1. Introduction

Tires equipped with different sensors have always been an area of research interest and ongoing progress [1]. Now with the emergence of automation, applications like self-driving cars, mobile robots and, most importantly, vehicle active safety systems expect tires to gather as much information as possible [2,3]. Adverse road conditions and faulty tires are often responsible for traffic accidents [1]. Therefore, sensors embedded in tires capable of monitoring real-time road conditions and tire health would be a step ahead in terms of automation and safety measures [4,5]. Additionally, in the development phase of a new tire, various conditions for tire testing need to be monitored and validated. A current method for developing new tires employs a pressure pad to measure tire mechanics, including tire tread (footprint) pressures, while the tire rolls through the pad [6]. This method does not provide real driving experience and also limits dynamic testing. Dynamic testing with different conditions is a major step while developing a new tire [7]. Sensor-embedded tires could improve the current tire testing system and provide opportunities for diverse testing conditions. Current sensors used in tire testing have limitations, including flexibility, stretchability, sensitivity, and stability [8,9,10], which is the motivation of this work.

To explore stretchable and flexible sensors, CNT/polymer composite-based sensors have been studied for quite a long time [11,12]. Many flexible pressure or strain sensors have been developed with CNTs, however, these sensors have limitations in terms of repeatability and stretchability [13,14]. Recently, with the development of ionic liquids (ILs), the area of pressure sensors has opened a novel window. ILs comprising of organic cations and counterions have been a subject of research interest in various areas because of their high ionic conductivity, negligible vapor pressure, low flammability, and electrochemical stability [15,16]. IL/polymer composites have also been studied, while they are developed by integrating ILs into prepolymers so that they, subsequently, become polymerized [15,17,18]. These IL/polymer composites have added a whole new dimension for stretchable and flexible pressure sensors. Using this composite material, multilayer stretchable sensors are fabricated and evaluated [19]. A unique feature of this IL-based stretchable sensor is that it provides a few degrees of freedom on controllable parameters, such as IL concentration, sensor geometry, degree of polymerization, etc., which can be chosen according to applications [20]. Several pressure sensors based on ILs have been also presented which are in a liquid state [21]. They have limited applications and drawbacks, such as leaking. Here, in this study, research has been continued on pressure sensors with the solid-state IL from previous work [19,20]. Another aspect of the sensors are the functional and stretchable materials which are compatible with 3D printing.

Due to the difference in the mechanical properties of soft matters and rigid electronics, incorporating electronic devices within highly-stretchable media is challenging and creates inconvenience. That is why soft electronics, in particular, soft sensors, have a potential range of application in soft objects. To produce soft electronics, a few manufacturing processes have been attempted, including screen printing [22,23,24,25], coating [26,27,28], micro-channel molding [29], and filling and lamination techniques [30,31]. However, these processes are limited in 3D designs and extensibility along with high cost, poor durability, lack of manufacturing scalability [32]. 3D printing in the field of soft electronics could be a vital player as it overcomes the limitations of traditional manufacturing processes [33,34]. 3D printing can fabricate any complex shape and provides scopes for conformal, free-form fabrication and customization [35,36]. It has more control in manufacturing which results in better performance of devices and reduces the wastage of materials. 

In this work we demonstrate a 3D-printed tire assembly with prepared pressure sensors, which have not been explored. Hybrid manufacturing techniques combining a molding and screen-printing process has been used for simple and flat sensors for this work, although when the need is to fabricate a complex sensor or to have a sensor on a conformal surface, 3D printing is believed to be the best manufacturing process. Tires and wheels were 3D printed for the experiment. Fabricated sensors in this work were highly sensitive and, when embedded in tires, they give a scope to collect various information, such as load, speed, force location, etc. The sensor response obtained can lead to a number of practical applications, like road condition and tire health monitoring, movement control, obstacle avoidance, etc. 

## 2. Design, Materials, and Methods

### 2.1. Sensor Design and Principle

**Figure 1:**
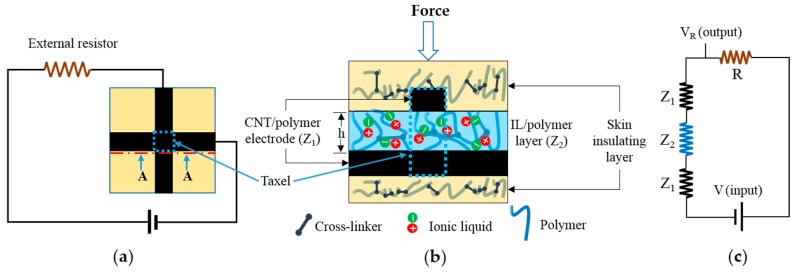
(**a**) A single taxel sensor connected to a half-Wheatstone bridge circuit supplied with the DC voltage; (**b**) detailed view of the cross-section of a taxel with different layers; and (**c**) a simplified equivalent circuit showing resistance of each layer.

The proposed flexible sensor consists of five layers. As illustrated in Figure 1b, top and bottom parts are the insulating skin-like layers, which act as an insulator. There are two layers of a multi-walled carbon nanotube (MWNT)/polymer composite which work as conductive electrodes. An IL/polymer composite layer is sandwiched between these electrodes. This intermediate layer is piezoresistive and contributes to the sensitivity of the sensor under force. The area where electrodes cross each other is the sensitive zone and called a taxel (each sensing unit). For this work two different sensors were used: one with six taxels providing six sensing units at different locations and another with twelve taxels.

Each taxel in the sensor was connected to a half-Wheatstone bridge circuit and voltage across an external resistor was measured to evaluate the response of the sensor under external force. Figure 1a shows a single taxel sensor connected to a circuit and supplied with DC voltage. Due to IL incorporation into a prepolymer matrix to fabricate the intermediate layer, ionic conductivity appears in that layer. This ionic conductivity develops from the transportation of ions between coordinate sites of the polymer chain under enough activation energy where the energy is provided by the external voltage source through creating a potential difference between the electrodes [16,19]. The sensor is deformed by external force, and the distance between electrodes, ‘h’ in Figure 1b, decreases. Moreover, the excitation in the IL/polymer piezoresistive layer occurs. These factors contribute in the drop of electrical resistance (Z_2_ in Figure 1c) of the intermediate layer, hence increase the voltage output across external resistor which is measured as output signal. The MWNT/polymer electrode can also be sensitive to pressure and can create crosstalk under deformation. To avoid that, a high loading ratio of MWNT (5 wt. %) was used to fabricate the electrode which is higher than the electrical percolation threshold of MWNT/polymer composites [20,37]. Electrical resistance of the carbon nanotube-based electrode was measured as 20 to 30 KΩ, whereas the IL-based intermediate layer has a resistance of a few hundred (200–300) MΩ when connected to the circuit. Thus, while maintaining a higher electrical conductivity in the MWNT electrode, its piezoresistive effect on the sensor is negligible. Resistance Z_2_ in Figure 1c is a few thousand times higher than resistance Z_1_ for the proposed experiments. V_R_ in Figure 1c is measured to calculate the sensor response. Further details for the sensor principle can be found in [19,20]. 

### 2.2. Materials 

Materials were prepared based on the prior study [19,20]. Top and bottom insulation layers were fabricated using a commercially available photopolymer (TangoPlus FLX930, Stratasys, Eden Prairie, MN, USA), which is 3D printable. The material for the stretchable electrode was prepared by dispersing 5 wt. % MWNTs (Nano Lab Inc., Waltham, MA, USA) into the TangoPlus FLX930. The specifications of the MWNTs are 5–20 μm in length, 10–30 nm in diameter, and over 85% pure. The process starts with dissolving a surfactant, Triton X100 (Sigma-Aldrich, Milwaukee, WI, USA), into dimethylformamide (DMF, Sigma-Aldrich). Then MWNT was added to the solution. The weight ratio of MWNTs to Triton X100 was taken as 1.0:3.5. The solution was sonicated (Q700, Qsonica, Newtown, CT, USA) for dispersion with a power of 700 W, a frequency of 20 kHz, and an amplitude of 50% for 20 min in pulse mode (1 min on, 10 s off). A magnetic stirrer was also used at the same time to create global agitation. After that, TangoPlus was added into the DMF/MWNTs solution and was mixed via agitation with the magnetic stirrer. The MWNTs/prepolymer was then kept on a hot plate magnetic stirrer (VWR 1010 ALU Hotplate, VWR, Radnor, PA, USA) at 80 °C and 400 RPM for 48 h to fully evaporate the solvent. Finally, the paste was mixed again using a high-speed mixer (DAC 150.1 FVZ-K, FlackTek, Inc., Landrum, SC, USA) at 2500 rpm for 1 h. To make the paste thermally curable, 5 wt. % of a thermal initiator (TRIGONOX 125C75, Akzo Nobel Functional Chemicals LLC, Chicago, IL, USA) was added and mixed again by the high-speed mixer for 5 min. To fabricate the IL/polymer layer 30 wt. % diluent, a photocurable monofunctional monomer (SR 278, Sartomer America, Exton, PA, USA) was mixed with TangoPlus. Finally, 3 wt. % ionic liquid, 1-ethyl-3-methylimidazolium tetrafluoroborate (EMIBF4, Sigma-Aldrich, St. Louis, MO, USA) was added to the mixture and blended using the high-speed mixer at 2500 RPM for 2 min. 

### 2.3. Fabrication of Sensors

**Figure 2:**
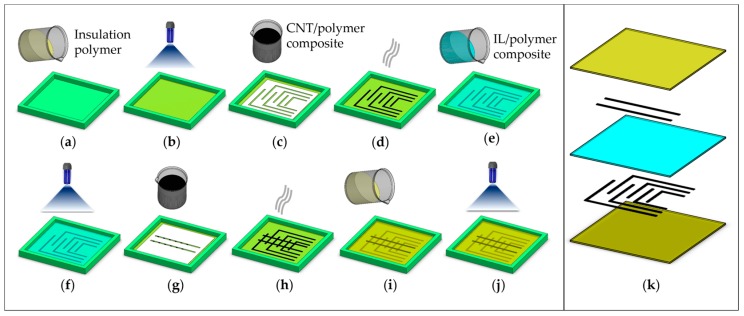
Sensor fabrication steps: (**a**) TangoPlus is poured into mold to create bottom insulation layer; (**b**) UV curing process for the bottom insulation layer; (**c**) screen printing of MWNT/polymer paste to create the first electrode layer; (**d**) thermal curing of MWNT/polymer electrodes; (**e**) IL/polymer composite poured into mold to create a piezoresistive intermediate layer; (**f**) UV curing of the IL/polymer layer; (**g**) screen printing of the MWNT/polymer paste to create a second electrode layer; (**h**) thermal curing of MWNT electrodes; (**i**) TangoPlus poured to create the top insulation layer; (**j**) UV curing of the top layer; and (**k**) an exploded view of sensor.

A hybrid manufacturing technique involving a molding and screen-printing process for a twelve-taxel sensor is illustrated in Figure 2. At first, the photopolymer TangoPlus was cast into a square mold (80 × 80 mm^2^) (Figure 2a) and then photocured (Figure 2b) using UV light (OmniCure^®^ S2000, Excelitas Technologies Co., Wheeling, IL, USA) to create the bottom insulation layer of 1.0 mm thickness. Then the first electrode layer with six separate electrodes was screen printed (Figure 2c) directly on the bottom insulation layer using the prepared MWNT/prepolymer composite paste. A mask was made from photo paper for screen printing using a Silhouette Cameo (Silhouette America Inc., Lindon, UT, USA) cutting machine. The printed MWNT/prepolymer electrodes had a width of 1.5 mm and a thickness of 200 μm. These electrodes were thermally cured (Figure 2d) at 80 °C for 5 min in an oven. The IL/polymer composite material was poured (Figure 2e) into the mold and polymerized (Figure 2f) using the UV light to create the piezoresistive layer. The thickness of the piezoresistive layer was 1.0 mm. The second electrode layer containing two electrodes of the same dimension was printed (Figure 2g) on the piezoresistive layer and cured (Figure 2h) with the same method for the first electrode layer. Finally, TangoPlus was poured (Figure 2i) and cured (Figure 2j) to create the top insulation layer of 1.0 mm thickness. The overlapped areas covered by both layers of electrodes work as a sensing unit and, thus, the sensor in Figure 2 has 12 taxels. Figure 2k shows an exploded view of the sensor with different layers.

### 2.4. Design of Tires, Wheels, and Chassis

**Figure 3:**
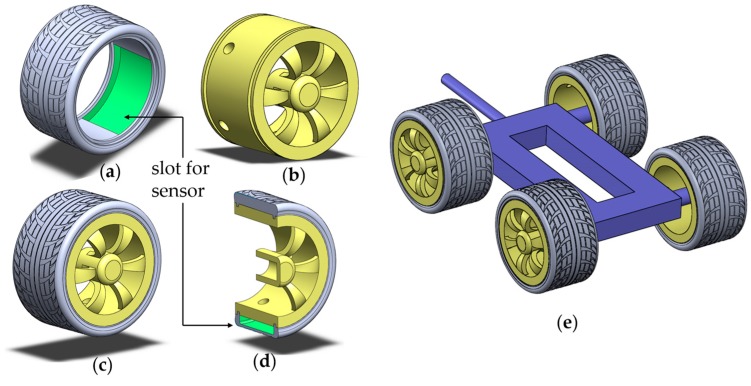
(**a**) Tire with slot for sensor; (**b**) wheel with hole for wiring; (**c**) tire-wheel assembly; (**d**) sectional view of the assembly; and (**e**) tire, wheel, and chassis assembled.

A tire model was designed for 3D printing having a slot in the inner surface to attach the sensor as shown in Figure 3a. The outer diameter of the tire is 120 mm and the width is 62 mm. The dimension of the slot to secure the sensors was 80 × 50 × 4 mm^3^. The wheel was designed accordingly. Some holes were kept on the wheel for wiring of the sensor, as illustrated in Figure 3b. Finally a chassis was designed having a 220 mm length and a 120 mm width. A shaft was designed on the chassis to attach it with the motorized linear stage. Assembly of all of these models are shown in Figure 3. The shaft diameter was 12.7 mm. These models were fabricated in commercial 3D printers.

### 2.5. Experiments

**Figure 4:**
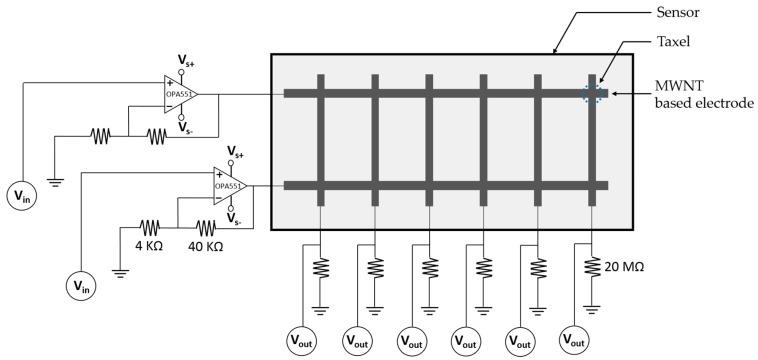
Wiring diagram of a twelve (2 × 6) taxel sensor where each taxel is connected to a half-Wheatstone bridge circuit. For the operational amplifier (OPA551PA), the supply voltage range was +24 to −24 V, and the input voltage range was +10 to −10 V.

The experimental setup was comprised of a data acquisition device (DAQ, BNC-2090A, National Instruments, Austin, TX, USA), external power supply (E3630A, Keysight Technologies, Santa Rosa, CA, USA), and motorized linear stage (A-LST250B-E01, Zaber Technologies, Vancouver, BC, Canada) with a resolution of 0.1 μm and a maximum travel range of 250 mm. The car chassis was connected to the linear stage firmly and the stage movement was controlled by Zaber Console software provided by the manufacturer of the stage. The DAQ was interfaced with MATLAB/Simulink and was used to measure the voltage output across the external resistor (20 MΩ) of the half-Wheatstone bridge circuit. Two sensors were fabricated for this study: one with six taxels (1 × 6) and the other with twelve taxels (2 × 6). The 6-taxel sensor was used for different load condition experiments and the 12-taxel sensor was used for different speed condition experiments. During experiments with a sensor each taxel was connected to the half Wheatstone bridge circuit. An op-amp (OPA551PA, Burr-Brown, product from Texas Instruments, Dallas, TX, USA) was used with the DC supply voltage of 24 V for each of the input electrode of the sensor. The wiring diagram of the twelve-taxel (2 × 6) sensor is shown in Figure 4. The input voltage in each electrode was controlled through DAQ. A low-pass Butterworth filter was used for the data received to reduce noise.

For the first set of experiments, the 6-taxel sensor was inserted inside the tire slot and connected to the circuit. The car was driven at 5 mm/s, 10 mm/s, and 50 mm/s speeds. For each speed the car chassis was loaded with 0.38 kg, 1.50 kg, and 5.00 kg weights and sensor responses were recorded. For the second set of experiments, the 12-taxel sensor was embedded in tire and the car was driven at 5 mm/s, 10 mm/s, and 50 mm/s speeds with the weight of 0.38 kg while the sensor responses were recorded as voltages. Additionally, the locations of the applied force were detected.

## 3. Results and Discussion

### 3.1. Manufactured Parts and Assembly

#### 3.1.1. Fabricated Sensor

**Figure 5:**
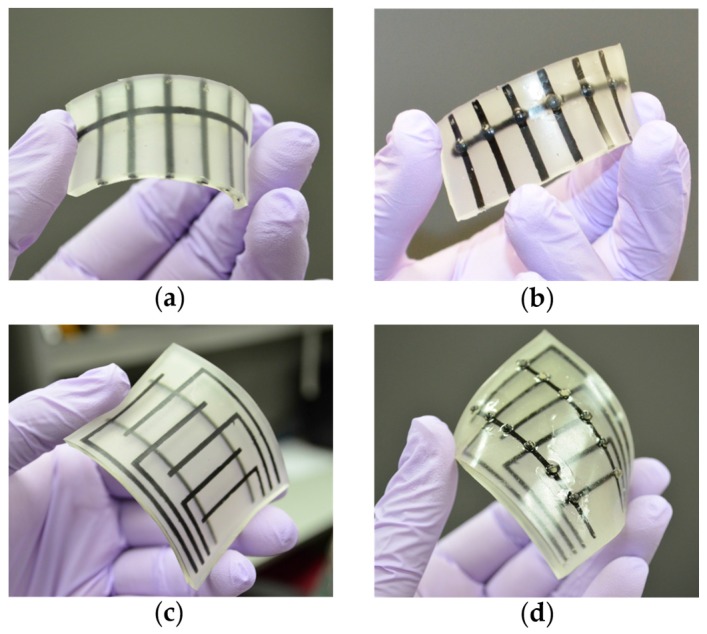
(**a**) Six-taxel (1 × 6) sensor; (**b**) 6-taxel sensor with bead; (**c**) 12-taxel (2 × 6) sensor; and (**d**) bead attached on each taxel.

Two sensors were fabricated with six (1 × 6) taxels and twelve (2 × 6) taxels. The 6-taxel sensor has six electrodes in one layer and one electrode in another layer. Dome-shaped small beads were attached on the taxel. To attach the beads, TangoPlus was brushed on the sensor, then the beads were put on taxels. Finally, TangoPlus was cured by UV light. Beads were used on the taxel to make the taxel more reachable and sensitive. Having the sensor inside the tire, force from outside distributes to a larger area, which makes the sensor less sensitive. This is the reason why the beads were attached to get higher pressure. Figure 5 shows the sensors with and without beads. Similarly, beads were added to the 12-taxel sensor, too. The thickness of the fabricated sensor was around 3 mm. Sensors can be made thinner or thicker according to applications.

#### 3.1.2. 3D Printed Tires, Wheels, and Chassis

**Figure 6:**
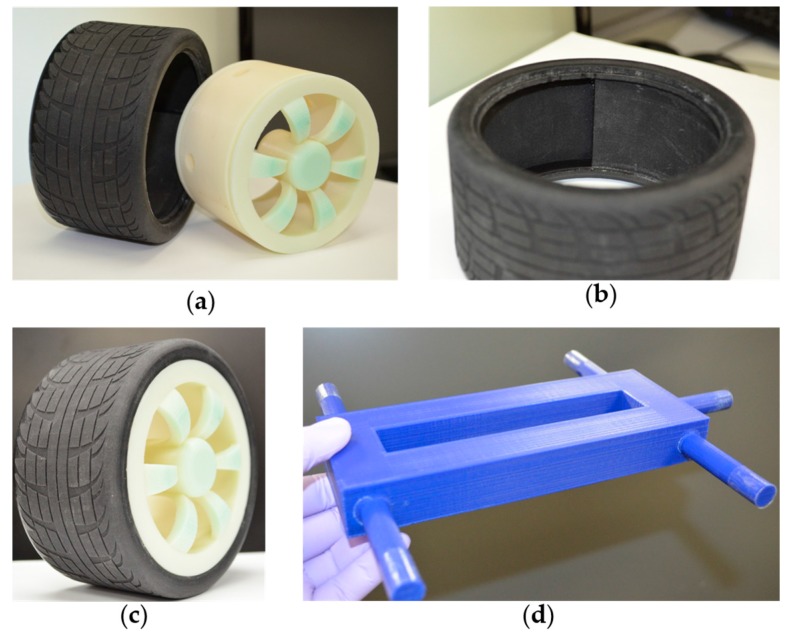
3D printed tire assembly. (**a**) 3D printed tire and wheel; (**b**) slot for the sensor inside the tire; (**c**) the assembled tire on the wheel; and (**d**) the 3D-printed chassis

Four tires were 3D printed using flexible material, TangoBlack, through a polyjet printer (Objet, Eden 260 V, Stratasys). The wheels were 3D printed using a rigid material, ABSplus thermoplastic, with an FDM 3D printer (uPrint SE Plus, Stratasys). The chassis was also printed using an FDM 3D printer (MakerBot Replicator). Total weight of the car assembly was 1630 gm (four tires, four wheels, chassis, and the sensor). Figure 6 shows the 3D-printed parts for the car.

#### 3.1.3. Assembly

**Figure 7:**
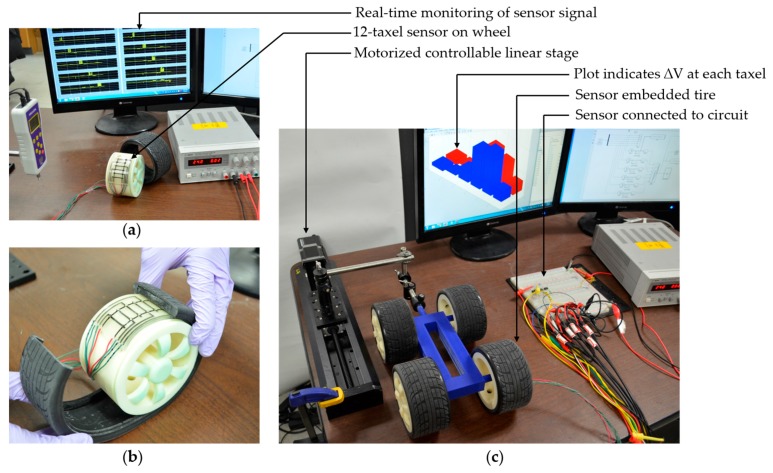
(**a**) Twelve-taxel sensor attached on the wheel; (**b**) sensor connected and inserted inside the tire; and (**c**) the fully assembled experimental setup with the motorized linear stage.

Figure 7 illustrates the assembly for the 12-taxel sensor experiments. The sensor was wired and securely mounted on the wheel while wires from the sensor come through the hole in the wheel. Common solid core electrical wire (copper) was pierced into the sensor to connect with MWNT-based electrodes. The tire was assembled carefully with the wheel so that the sensor goes into the slot inside the tire. Only one tire was equipped with the sensor. The whole car assembly was firmly connected to a high-resolution motorized linear stage to control the speed of the car. Figure 7c shows the assembly and wiring of the system. 

### 3.2. Experimental Results

Experiments were done in two phases with two different sensors. First, different load conditions at various speeds were tested with the six-taxel sensor. Second, the twelve-taxel sensor was inserted inside the tire and driven at different speeds with the load of 0.38 kg. Speed was calculated from the sensor data and compared with the original speed from the stage. Additionally, detection of force location was shown. 

#### 3.2.1. Different Load Conditions

**Figure 8:**
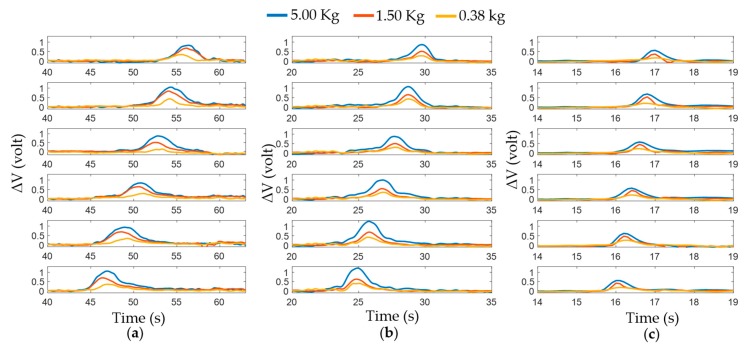
Changes in voltage output versus time for different load conditions: Voltage output with the speed of (**a**) 5 mm/s; and (**b**) 10 mm/s; (**c**) 50 mm/s

The car was driven at 5 mm/s, 10 mm/s, and 50 mm/s speeds for this experiment. For each speed the car was mounted with a load of 0.38 kg, 1.50 kg, and 5.00 kg. Figure 8 illustrates a graph indicating the change in voltage output versus time to show the sensor response at different conditions. As the tire rotates, the taxels come in contact with the road one by one. Once a taxel comes down to the road, the weight of the car creates compressive strain on the taxel, which results in a decrease in sensor resistance. Thus, the voltage output increases across the external resistor of the half Wheatstone bridge circuit. Changes in voltage output are indicative of the external force applied on the sensor. As expected, Figure 8 shows higher voltage increase for the higher load. The blue line on top indicates a 5 kg load and a yellow line at the bottom denotes a 0.38 kg weight. Additionally notable is that the taxels are showing a response at different times according to speed. Comparing Figure 8a–c, it is seen that, for the same weight, voltage change varies with speed. The difference is not that significant between 5 mm/s and 10 mm/s, but it is clear between 5 mm/s and 50 mm/s or 10 mm/s and 50 mm/s. For the higher speed changes in voltage are comparatively lower which may come from the shorter time period for the normal force to act on the taxel. 

#### 3.2.2. Location and Speed

**Figure 9:**
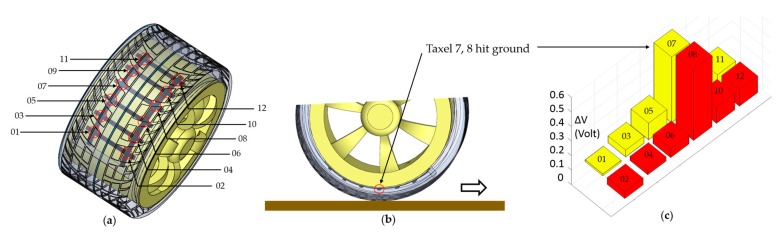
(**a**) Twelve locations on the tire were marked corresponding to 12 taxels; (**b**) the tire rotates while taxels or location 7 and 8 hit the ground; and (**c**) bar plot indicates ∆V at each taxel when locations 7 and 8 hit ground.

A twelve-taxel sensor was embedded in the tire. Each taxel is attached and corresponds to a unique location on the tire, as shown in Figure 9a. To graphically represent these twelve locations in the tire and force at each location, a bar plot was drawn. Each bar in Figure 9c indicates a certain taxel and a certain place in tire. Once the tire rotates, the bar plot shows the voltage change in each taxel according to the force upon it. For example, Figure 9c shows ∆V at a certain time. Bars 7 and 8 show the highest peak, which means locations 7 and 8 undergo weight/force at that certain point of time. Figure 9b is the corresponding tire situation when it rotates forward.

**Figure 10:**
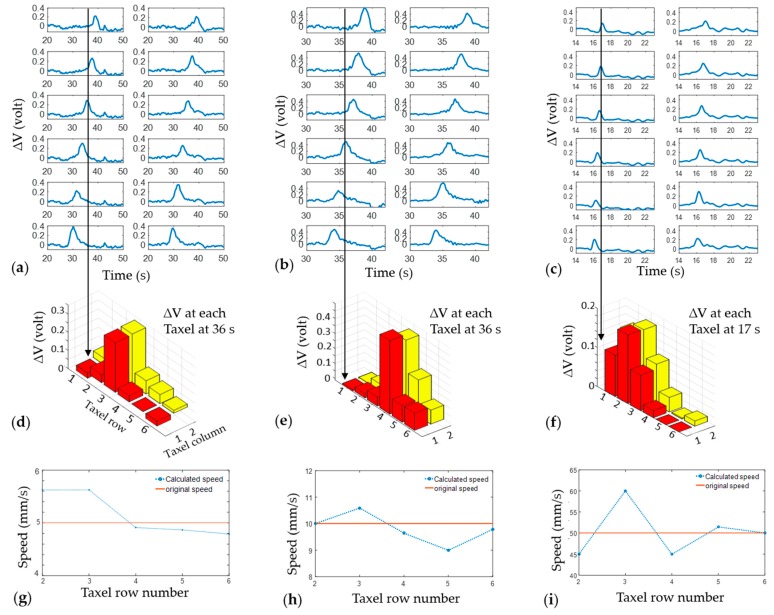
The car was loaded with a 0.38 kg weight and experimented at different speeds: the change in voltage output versus time for 12-taxel sensor embedded in tire while car speed was (**a**) 5 mm/s; (**b**) 10 mm/s; and (**c**) 50 mm/s. (**d**–**f**) The locations of force shown in the bar plot at a certain time. (**g**–**i**) The speed of the car calculated at each row of taxel and compared with original speed.

The speed of the car was measured from the sensor data while the car was loaded with 0.38 kg of weight. Taxels are 9 mm away from each other in the longitudinal direction and the two farthest taxels have a distance of 45 mm between each other. Figure 10 shows the experimental results for three different speeds. From the pick of the voltage change, the time to hit each taxel was measured and using this time and distance between the taxels, the speed was calculated. Speed can be measured at each row of taxels, from the second to the last row. With six rows of taxels, speed was calculated five times and compared with the original speed provided in the stage. The calculated speed was close to the original speed. Some deviation can occur due to friction between the chassis shaft and the wheel bore. No bearings were used for these experiments and some frictional resistance was noticed between the chassis shaft and the wheel bore. Locations of force are also shown at certain times in Figure 10d–f.

### 3.3. Discussion

#### 3.3.1. Result Analysis

As a whole, the experimental results have shown a trend throughout the work. However, there are some cases of disturbed outcomes. In particular, the speed calculated in Figure 9i shows a large deviation from the original speed provided to the stage. There could be a number of reasons behind this deviation. Due to the lack of any bearing, there was friction between the chassis shaft and wheel bore. There were vibrations while the car was moving and the direction of the movement could also change slightly. Thus, using bearings could improve the experimental setup considerably. The electrical noise in the voltage output signal was at a reasonable level and signal peaks due to force on the sensor were distinct from noise. The signal-to-noise ratio (SNR) of the output voltage was calculated using a MATLAB function (snr), with a result of 37.28 dB. However, in some cases, electrical noise has been noticed (such as Figure 9a). Better filtering could reduce the noise in the voltage output data received through the DAQ. In upcoming works an improved electrical noise reduction system, as well as bearings for the car, will be used for obtaining better data. Different taxel data (voltage output) of the sensor could be slightly non-uniform because of manufacturing variables and artifacts. However, each taxel can be calibrated separately for force to maintain consistent results.

To verify the effect of bending curvature on the sensor, the sensor was tested by keeping it on a flat surface and a curved wheel. There was a negligible difference in the signal output while normal force was applied to the sensor, which is the dominant factor in the change of voltage output. Moreover, the sensor could be calibrated to measure force once it is mounted on the wheel so that there will be no effect of curvature. Another area of limitations was the distance travelled by the car. Due to the short range of the stage, the maximum distance the car could travel was 250 mm. Additionally, wires from the sensor to the circuit board restricted the longer run of the car. As a result of these limitations, the tire could not rotate more than once in a single run. PCB-embedded sensors with wireless signal monitoring are within the scope of future research. In addition, a robotic car can be used with changeable tires. 

#### 3.3.2. 3D Printing Prospect of Sensor

**Figure 11:**
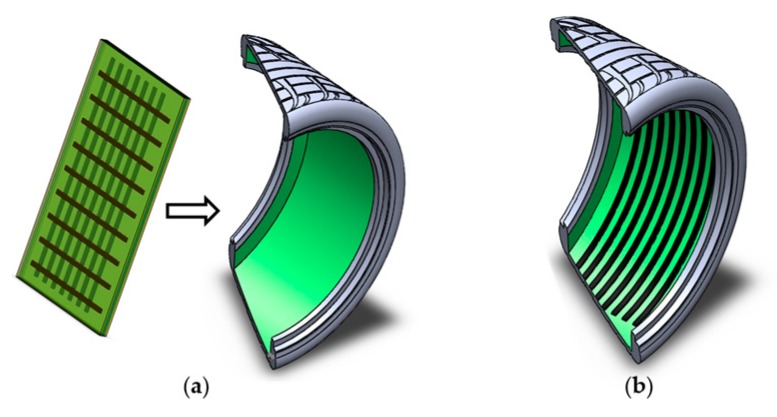
(**a**) Conventional sensor installation on tire; and (**b**) proposed direct-print photopolymerization of MWNT-based electrodes on the conformal inner liner surface of the tire.

3D printing is believed to be the most suitable manufacturing process of the pressure sensor for conformal surface applications. If a sensor is to be embedded in a freeform or conformal surface, 3D printing can overcome the margins of traditional processes. For the case of the tire sensor, we have the conformal shape of the inner liner surface. Figure 11a shows conventional installation process of the sensor on the tire, which is limited in terms of manufacturing scalability and design flexibility. 3D printing of the sensor on the inner tire surface could give a “print-it-all” solution, as shown in Figure 11b. A direct-print photopolymerization (DPP) process, which has been recently developed by the authors’ group is being investigated to print the sensor on the tire surface [38]. Conformal direct-print/cure has been reported in earlier work [39] with a different type of sensors. The system comprises of three high-resolution stages and a direct-cure unit with a UV lamp with optical fibers. A multilateral direct-print system is under development and research on the DPP of the IL-based sensors on the conformal tire surface is ongoing. 

## 4. Conclusions

In this work, flexible multi-taxel piezoresistive sensors have been demonstrated for tire applications. Material preparation and manufacturing techniques of the sensor were reported. The sensor was embedded on the inner surface of the tire for tire condition experiments. A series of experiments was accomplished to test different load and speed conditions. With the increase of weight, change in voltage output (∆V_out_) increases, which is indicative of a higher force on the tire. By analyzing each taxel data separately, the location of forces was detected, and the speed of the car was calculated. The important information extracted from the sensor response, such as load, speed, location, etc., could be valuable for many automation applications. Mobile robots and self-driving cars could be potential fields of sensor embedded tires. In the future, scopes of the experiments will be broadened with more taxels in sensors and different road conditions will be tested. In terms of manufacturing the sensor, 3D printing is our goal, where all of the materials used for this work are 3D printable. 3D printing of sensors on a conformal tire surface is another research focus for the future. This work is the first step to realize our objective. Our final goal includes fully-3D-printed parts with embedded wireless devices to transmit sensor signals. A wireless sensor system in a tire would overcome the limitation of wiring. We are also planning to operate, in the next work, a motorized car to expand the area of experiments.
